# Interplay of Na Substitution in Magnetic Interaction and Photocatalytic Properties of Ca_1‐x_Na_x_Ti_0.5_Ta_0.5_O_3_ Perovskite Nanoparticles

**DOI:** 10.1002/open.202400021

**Published:** 2024-08-30

**Authors:** S. S. Kammar, V. K. Barote, A. A. Gaikwad, Sagar E. Shirsath, A. A. Ibrahim, K. M. Batoo, R. H. Kadam, S. S. More

**Affiliations:** ^1^ Department of Physics HKE's A. V. Patil Degree College Aland Kalburgi, KA India; ^2^ Department of Physics Sant Dnyaneshwar Mahavidyalaya Soegaon 431120 Maharashtra India; ^3^ People's Education Society' College of Engineering Aurangabad 431001 Maharashtra India.; ^4^ Department of Physics Vivekanand College Aurangabad 431001, MS India; ^5^ School of Materials Science and Engineering University of New South Wales Sydney NSW 2052 Australia; ^6^ Department of Physics and Astronomy, College of Science King Saud University P.O. Box−2455 Riyadh 11451 Saudi Arabia; ^7^ King Abdullah Institute For Nanotechnology King Saud University P.O. Box−2455 Riyadh 11451 Saudi Arabia; ^8^ Materials Science Research Laboratory Shrikrishna Mahavidyalaya Gunjoti Osmanabad, MS India; ^9^ Department of Physics Y. C. College Tuljapur Osmanabad, MS India

**Keywords:** Sol-gel method, X-ray diffraction, magnetization, photocatalytic properties

## Abstract

This research paper delves into the enhancement of wastewater treatment through the design and synthesis of advanced photocatalytic materials, focusing on the effects of sodium (Na) substitution in Ca_1‐x_Na_x_Ta_0.5_Ti_0.5_O_3_ perovskites. By employing various analytical techniques such as X‐ray diffraction, Field Emission Scanning Electron Microscopy, Transmission Electron Microscopy and UV‐vis spectroscopy, the study examines the transition of these perovskites from tetragonal to orthorhombic structures and observes a reduction in Ca content with Na substitution, which also favors the cubic phase formation and inhibits secondary phases. Significantly, magnetic property analysis uncovers an unexpected ferromagnetic ordering in these perovskites, including compositions traditionally viewed as non‐magnetic. The photocatalytic tests reveal a significant improvement in degrading Rhodamine B dye under visible light, particularly in samples with higher Na levels, attributed to enhanced light absorption and efficient electron processes. The study highlights the optimal Na substitution level for peak photocatalytic performance, offering valuable insights into the complex interplay between structural, magnetic, and photocatalytic properties of these perovskites, and their potential in various applications, thereby contributing to the advancement of wastewater treatment technologies.

## Graphical abstract

Photocatalytic process with a scene of water purification and environmental enhancement using Na substituted Ca_0.2_Ta_0.5_Ti_0.5_O_3_ perovskite nanoparticles. The specific composition, Ca0.2Na0.8Ta0.5Ti0.5O3, exhibits enhanced photocatalytic activity, as demonstrated by reflectance spectra showing bandgap energy and kinetics graphs of photocatalytic degradation. This substitution tailors the material for more effective environmental purification.

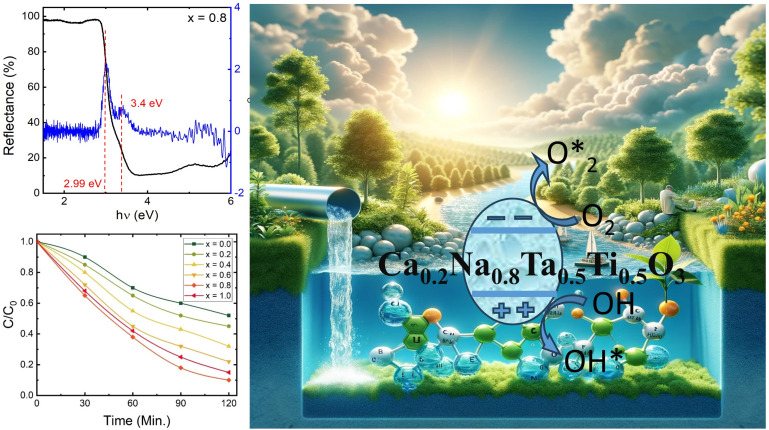

## 1 Introduction

Addressing the substantial wastewater produced by swift industrial and urban growth presents a major worldwide dilemma. The situation worsens because traditional treatment techniques – mechanical, physical, chemical, and biological – fail to fully degrade many pollutants, leaving behind substances that are tougher to break down. These conventional methods, while effective in removing a large portion of pollutants, still leave behind a fraction of contaminants that are more resistant or “refractory” in nature. As a result, there is a pressing need to develop more economical and efficient methods to treat these stubborn pollutants.

Semiconductor photocatalysis emerges as a promising solution in this context. This advanced oxidation technology leverages light‐activated materials, known as photocatalysts, to generate highly reactive radicals. These radicals are effective in breaking down and completely mineralizing the adsorbed pollutants, essentially turning these harmful substances into harmless inorganic compounds like water and carbon dioxide.[^1^, ^2^, ^3^] A key advantage of semiconductor photocatalysis over traditional wastewater treatment methods is its ability to deal with these refractory pollutants without producing secondary pollution. Traditional methods often result in secondary waste or by‐products that require further treatment, whereas semiconductor photocatalysis aims for complete mineralization of pollutants. The efficiency of photocatalysis is largely dependent on the performance of the photocatalyst. A high‐performance catalyst can significantly improve both the rate and the selectivity of the chemical reactions involved. This means pollutants can be degraded more quickly and selectively, minimizing the possibility of incomplete breakdown or the formation of intermediate harmful substances. The development of suitable photocatalysts is therefore a critical area of research and development in semiconductor photocatalysis. The ideal photocatalyst should be highly efficient, stable under various environmental conditions, and responsive to a broad spectrum of light, including sunlight. Current research is focused on finding materials and developing techniques that can enhance the photocatalytic activity of these catalysts. This includes doping with various elements, creating composites with other materials, and engineering the catalysts at the nanoscale to increase their surface area and reactivity.

The photocatalytic process in semiconductor photocatalysts is fundamentally reliant on the generation and behaviour of photogenerated electrons (e^−^) and holes (h^+^). These electrons and holes are crucial for initiating the photocatalytic reactions that lead to the degradation of pollutants. However, one of the primary challenges in this process is the tendency of these photoexcited electron‐hole pairs to recombine quickly, which reduces the number of pairs available for effective photocatalytic reactions. For a semiconductor photocatalyst to be efficient, it is crucial to enhance the separation and reduce the recombination of photoexcited electron‐hole pairs. This improved separation amplifies the availability of electrons and holes for the crucial oxidation and reduction reactions that decompose pollutants. Titanium‐containing oxide semiconductors stand out as a notable group within this context. These materials are particularly interesting due to their photocatalytic activity under ultraviolet (UV) light, a property attributed to their wide bandgap (Eg=3.1–3.3 eV).[^4^, ^5^, ^6^] However, a limitation of these semiconductors is their dependency on UV light for activation. Considering that only about 5 % of solar radiation is in the UV spectrum, with a much larger portion (around 45 %) being visible light, the reliance on UV light restricts the utilization of solar energy in photocatalysis. Therefore, enhancing the visible‐light absorption capabilities of photocatalysts is crucial for more effectively harnessing solar energy. By increasing a photocatalyst‘s ability to absorb visible light, more electron‐hole pairs can be generated using the solar spectrum, thereby enhancing the overall efficiency of the photocatalytic process. To address this, various strategies have been developed to modify semiconductor photocatalysts. These modifications aim to both facilitate the separation of photoexcited electron‐hole pairs and expand the range of light absorption, particularly into the visible spectrum. Till date, various strategies have been widely applied to modify semiconductor photocatalysts with the aim of facilitating the photoexcited e^−^/h^+^ pair separation and widening their light absorption range.[^7^, ^8^, ^9^, ^10^, ^11^]

Nanomaterials known to exhibit novel properties resulted into their applications in many technological fields.[^12^, ^13^, ^14^] Thus in turns, nanomaterials are effectively utilized as modifiers or co‐catalysts, often combined with semiconductors to boost their efficiency in photocatalysis.[^15^, ^16^, ^17^] Among these, noble metal nanoparticles (NPs) stand out as co‐catalysts for their dual function: they not only aid in separating photoexcited electron‐hole pairs but also increase the absorption of visible light. The improvement in photocatalytic activity due to noble metal NPs is primarily attributed to two factors.^[18]^ Firstly, these NPs serve as electron traps, capturing electrons generated by the semiconductor. This action leads to a more effective separation of electron‐hole pairs. Secondly, noble metal NPs can absorb visible light, initiating localized surface plasmon resonance (LSPR).^[19]^ The electromagnetic field produced by LSPR enhances both the creation and division of electron‐hole pairs within the semiconductor. Additionally, the electrons excited in the noble metal NPs by LSPR contribute to the photocatalytic reactions. Owing to these distinctive characteristics, research has shown that semiconductors adorned with noble metal NPs exhibit a marked improvement in photocatalytic performance compared to their unmodified counterparts.[^8^, ^16^, ^17^, ^18^] Oxide nanomaterials known to exhibit unique properties with wide range of applications.[^20^, ^21^, ^22^, ^23^, ^24^] Oxide based perovskite materials, with a wide bandgap and suitable electrochemical potential, are the preferred choice.^[25]^ In recent years, the AMO_3_ family of perovskites (M: transition metal and A: rare earth) have been extensively investigated because of their significant physical properties like; optics, dielectrics, electrical and magnetic characteristics.[^26^, ^27^, ^28^, ^29^] In order to understand the magnetic characteristics in the presence of external perturbations in perovskites with the formula R_1‐x_A_x_Mn_1‐y_M_y_O_3_ (R or A: alkaline‐earth/rare‐earth) have been studied.^[30]^ Among the many perovskite systems; CaTaO_3_ and CaTiO_3_ are the promising perovskite materials with their interesting electric and magnetic properties. CaTiO₃ is an orthorhombic perovskite crystallizing in the orthorhombic Pnma space group with lattice parameters of: a=5.41, b=5.51 and c=7.69 Å. CaTiO_3_ is an n‐type semiconductor with an ABO_3_ perovskite structure, with Ti–O terminations and a band gap of 3.5 eV.[^31^, ^32^, ^33^] CaTiO_3_has also been prepared for photocatalytic purposes with the addition of lattice and surface modifiers.^[34]^

Bassekhouad et al. reported that the half‐life of the pollutant benzamide decreased from 80 min for the undoped sample to nearly 20 min for 1 % Na doped TiO_2_ prepared by impregnation.^[35]^ There are reports on improved DSSC efficiency of Na doped nanorods synthesized by hydrothermal method and better photoelectrochemical performance of Na doped TiO_2_ nanoflakes prepared by hydrothermal treatment of Ti foils.[^36^, ^37^] As per these reports, the incorporation of alkali metal cations like sodium in the TiO_2_ lattice play a vital role in tailoring the bandgap energy and improving the photoresponse of the material.^[38]^

Considering the interesting properties of CaTaO_3_ and CaTiO_3_ we have prepared a combination of these two‐perovskite structure with a stoichiometric chemical formula CaTa_0.5_Ti_0.5_O_3_. Furthermore, Na is substituted for Ca and studied the structural, magnetic and photocatalytic properties of Ca_1‐x_Na_x_Ti_0.5_Ta_0.5_O_3_ (x=0.0, 0.2, 0.4, 0.6, 0.8, 1.0). The novelty of the research lies in the development of a hybrid photocatalyst combining CaTaO_3_ and CaTiO_3_ perovskites with sodium substitution for calcium, aimed at enhancing photocatalytic efficiency for wastewater treatment by improving light absorption and electron‐hole separation.

## Methods and Materials

Perovskite samples of Na‐doped Ca_1‐x_Na_x_Ti_0.5_Ta_0.5_O_3_, with x values ranging from 0.0 to 1.0, were prepared using the sol‐gel auto‐combustion method.[^39^, ^40^, ^41^] Starting with high‐purity (>99 %) AR grade metal nitrates for each component, these materials were precisely measured based on stoichiometric ratios and dissolved in double‐distilled water, ensuring thorough mixing by stirring continuously for 30 minutes. To this solution, citric acid was introduced as a chelating agent at a 1 : 3 weight ratio to the metal nitrates, enhancing the complexation process. The mixture was then subjected to uniform heating on a magnetic stirrer equipped with a hot plate, maintained at a constant temperature of 90 °C. During this phase, liquid ammonia was judiciously added to the mixture to adjust the pH level to a neutral point of 7, while stirring and heating were sustained to promote homogeneity. This meticulous procedure led to the gradual thickening of the solution, culminating in its transformation into a gel‐like substance. In an exothermic reaction, the gel spontaneously combusted, resulting in the formation of brown ash. This ash was then meticulously ground, and the resultant material was subjected to a sintering process at 900 °C for an extended duration of 6 hours, producing fluffy samples. These samples were then pulverized into fine powders through an additional grinding step lasting 30 minutes, preparing them for a series of detailed characterization analyses to evaluate their properties and potential applications.

Room‐temperature powder X‐ray diffraction (XRD) patterns were recorded for all samples, scanning from 20° to 70° using Cu−Kα radiation with a wavelength of 1.5405 Å. The XRD patterns were obtained using a Bruker D8 Advance – AXS X‐ray diffractometer, operating at 360 V and 5.5 A and equipped with a nickel filter. Scanning electron micrographs of select samples were captured using a ZEISS (SUPRA 55) microscope, while transmission electron micrographs were acquired with a high‐resolution TEM microscope (Philips) at an accelerating voltage of 300 kV. The magnetic properties of the samples were analyzed using a vibrating sample magnetometer, measuring responses to an applied magnetic field of up to 15,000 Oe.

## 2 Results and Discussion

**Figure 1:**
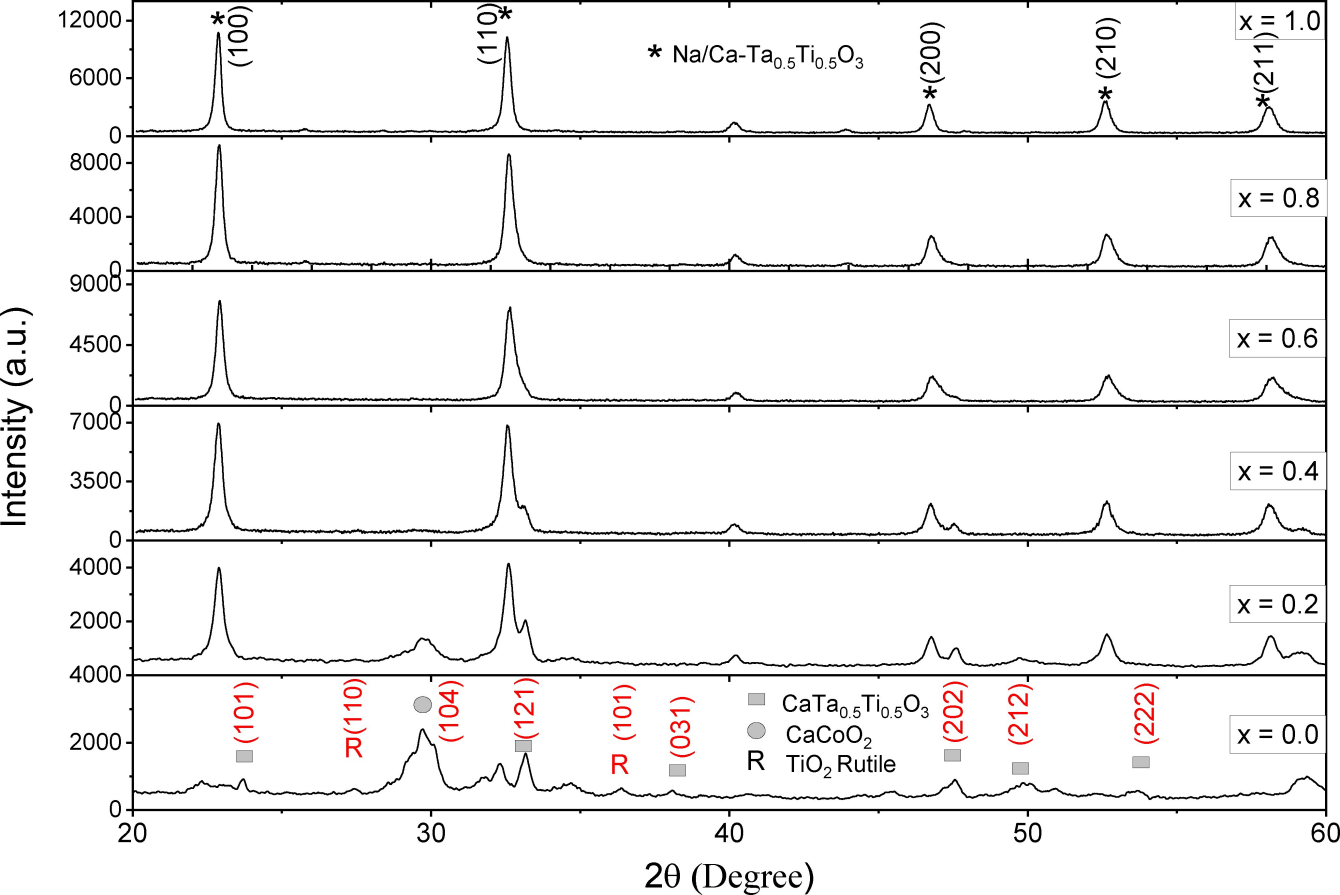
XRD patterns of all the samples of Ca_1‐x_Na_x_Ti_0.5_Ta_0.5_O_3_ (x=0.0, 0.2, 0.4, 0.6, 0.8, 1.0).

Figure 1 presents the X‐ray diffraction (XRD) spectra for a sequence of Ca_1‐x_Na_x_Ti_0.5_Ta_0.5_O_3_ perovskite compounds, where x varies from 0.0 to 1.0. The observed diffraction patterns correspond to the orthorhombic phase of CaTiO_3_, consistent with the standard reference pattern provided in the JCPDS card No. 42–0423,^[42]^ and the CaTa_0.5_Ti_0.5_O_3_ perovskite phase. A noticeable transition is observed from the original tetragonal TiO_2_ (rutile) structures to an orthorhombic CaTa_0.5_Ti_0.5_O_3_ structure.^[43]^ The rutile structures in the TiO_2_ precursor are identified according to JCPDS file Nos. 21–1272 and 21–1276.^[44]^ The patterns also reveal some background noise, likely stemming from the nanocrystalline nature of particles created through the sol‐gel method. A distinct peak around 29.35° suggests the presence of unreacted CaCO_3_ in the synthesis. The Ca/Ti ratio is typically greater than one in CaTiO_3_ based perovskites, likely due to cation non‐stoichiometry.^[45]^ Substitution of Ca by Na results in a decrement of the Ca concentration, thereby inhibiting the emergence of secondary phases. For substitution levels (x) equal to or exceeding 0.4, the cubic phase of CaTa_0.5_Ti_0.5_O_3_ becomes increasingly dominant over the orthorhombic phase, indicating a preferential stabilization of the cubic phase upon Na incorporation into the CaTiO_3_ lattice. At a substitution level of x=1.0, the lattice constants converge to approximately a=b=c=3.901 Å, underscoring the establishment of a cubic symmetry.

The apparent or experimental density (d) of a substance is calculated using pellets shaped as circles. This density determination involves measuring the mass (m) of the pellet, its radius (r), and its thickness (h). From these measurements, the volume (v) of the pellet is derived. With these parameters, the density can be accurately:^[46]^

(1) $$d=\frac{m}{v}=\frac{m}{\pi r^{2}h}$$

The experimental densities of the pellet samples showed a declining trend from 4.27 g/cm^3^ (for x=0.0) to 4.07 g/cm^3^ (for x=1.0), as Na was substituted in the composition. The specific surface area (S) of the samples was calculated using an equation that incorporates ′d′ and ′t′, representing the average crystallite diameter. This approach facilitated an accurate determination of the surface area‐to‐volume ratio of the crystallites:^[47]^

(2) $$S=\frac{6000}{t\times d}$$

The specific surface area of the samples exhibited an increase from 42 m^2^/g (for x=0.0) to 53 m^2^/g (for x=1.0) as a result of the sodium substitution. This change in both the apparent density and specific surface area correlates with alterations in particle and grain size caused by the introduction of sodium into the CaTa_0.5_Ti_0.5_O_3_ perovskite structure. Lattice constant, experimental/apparent density and surface area are comprehensively detailed in Table 1.

**Table 1:** Structural parameters, lattice constants (a), experimental/apparent density (d), specific surface area (S) of Ca_1‐x_Na_x_Ta_0.5_Ti_0.5_O_3_. Values in the square bracket present the estimated error.

Composition	a (Å) [±0.002]	d (g/cm^3^) [±0.1]	S (m^2^/g) [±2]
x=0.0	3.890	4.27	42
x=0.2	3.893	4.22	45
x=0.4	3.895	4.19	47
x=0.6	3.897	4.15	49
x=0.8	3.899	4.11	51
x=1.0	3.901	4.07	53

**Figure 2:**
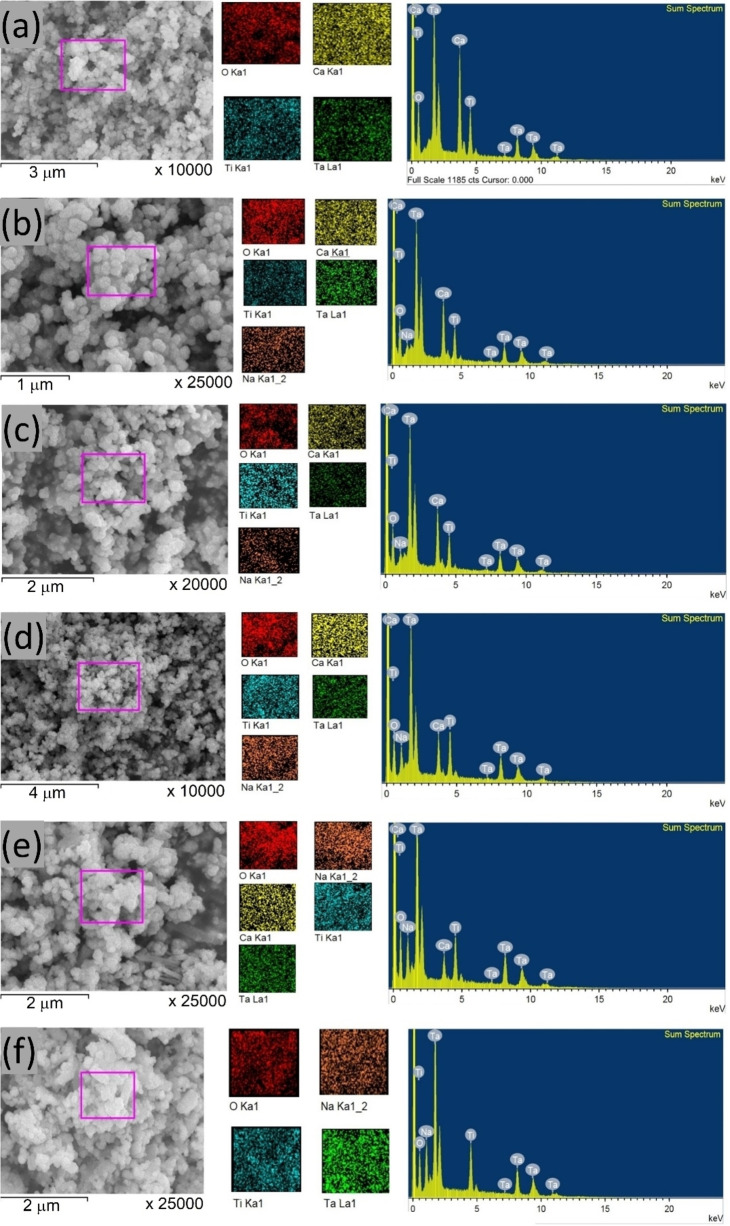
SEM images of Ca_1‐x_Na_x_Ta_0.5_Ti_0.5_O_3_, where (a) x=0.0, (b) x=0.2, (c) x=0.4, (d) x=0.6, (e) x=0.8 and (f) x=1.0.

Field emission scanning electron microscopy (FE‐SEM) micrographs showcasing Ca_1‐x_Na_x_Ta_0.5_Ti_0.5_O_3_ are displayed in Figure 2. These micrographs reveal that each sample comprises a mixture of nanocrystallites. The SEM images also show regular grain growth, which is slightly impacted by the Na substitution. The particles predominantly exhibit a spherical shape, although some elongated particles are also visible. Dense clusters are discernible in these images, suggesting a densification in the structure. The microstructure appears to be enhanced with the substitution of Na^2+^ ions, as indicated by the SEM images. This substitution leads to a slight decrease in the average grain size, from ∼90±2 nm (for x=0.0) to ∼80±2 nm (for x=1.0).

**Figure 3:**
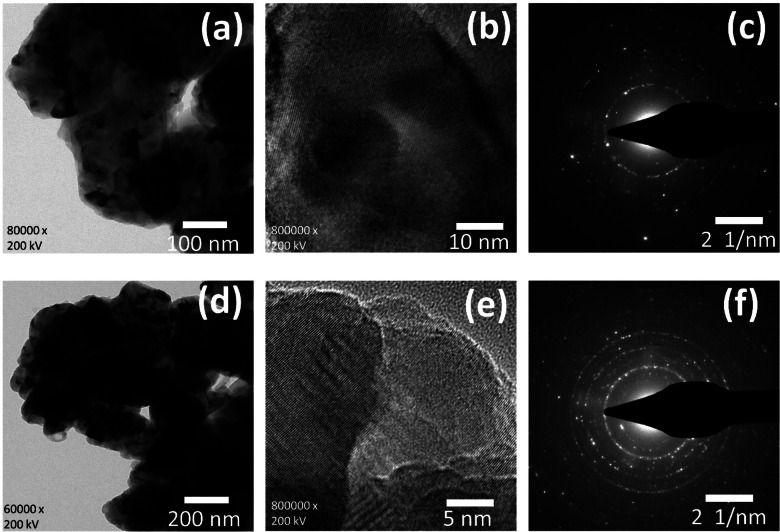
(a) and (d) are the TEM images of x=0.2 and x=0.8, respectively, (d) and (e) are the high‐resolution images of x=0.2 and 0.8, respectively, (c) and (f) are the corresponding SAED patterns of x=0.2 and x=0.8, respectively.

Figure 3 showcases TEM images along with selected area electron diffraction (SAED) patterns for specific samples with x values of 0.2 and 0.8. These TEM images reveal a significant agglomeration of particles. From these images, the average particle size, denoted as ′d′, is determined to be approximately 90±2 nm for x=0.2 and around 80±2 nm for x=0.8. The SAED patterns exhibit Debye ring patterns, which are indicative of the polycrystalline nature of these samples. Notably, the ′d′ values obtained from the SAED patterns are in good agreement with those derived from the XRD patterns, further confirming the consistency and reliability of the measurements across different analytical techniques.

**Figure 4:**
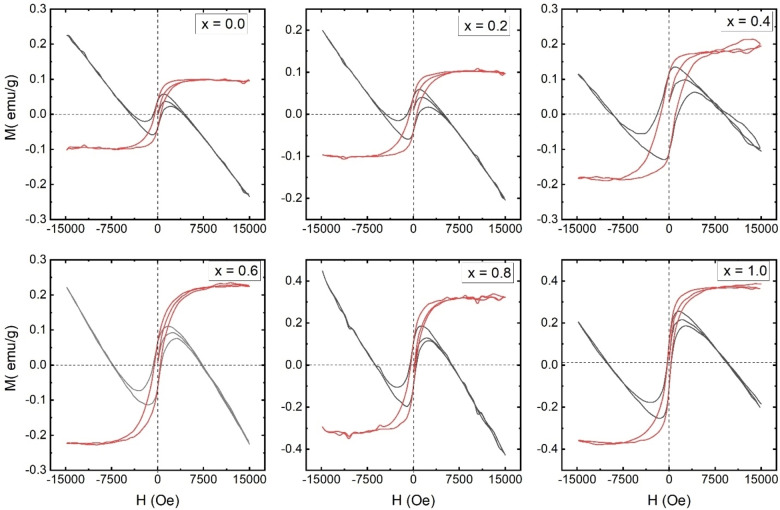
Magnetization ‘M’ as a function of applied magnetic field ‘H. Plots in grey color are as‐received plots having the diamagnetic contribution. MH plots in red color are hysteresis loops after removing the diamagnetic contribution.

In Figure 4, the magnetization (M) behaviour of Ca_1‐x_Na_x_Ta_0.5_Ti_0.5_O_3_ perovskite samples is illustrated as a function of the applied magnetic field (H), depicted through M−H loop measurements. These loops were analysed to determine key magnetic properties, such as saturation magnetization (Ms) and coercivity (Hc). The M−H loops exhibit an ′S′ shape, confirming the presence of ferromagnetic ordering in all Ca_1‐x_Na_x_Ta_0.5_Ti_0.5_O_3_ samples. The magnetization shows a rapid increase with the application of the magnetic field and reaches saturation at approximately 5 kOe. Interestingly, there is a decline in magnetization at higher magnetic fields beyond 10 kOe. This decrease might be attributed to a small amount of diamagnetic phase present in the samples, which becomes more prominent at higher magnetic fields.

Saturation magnetization is observed to increase with the substitution of Na, as shown in Figure 4. The magnetic moment is primarily a result of unpaired electron spins. According to Neel's model, the magnitude of magnetization (M) can be inferred by considering the distribution of metal ions and the antiparallel spin arrangement on two sub‐lattice sites. Neel's model predicts three types of magnetic interactions: A−A, A−B, and B−B, with A−B super‐exchange interaction being the strongest. Magnetization is thus determined by the vector sum of the magnetizations of the two sub‐lattices, i. e. Ms=MB ‐ MA, where MA and MB represent the magnetizations of the A and B sub‐lattices, respectively.[^48^, ^49^, ^50^]

It′s noteworthy that NaTiO_3_ is known for its ferromagnetic properties with a magnetic moment of 0.99 μB/f.u. which contributes to higher magnetization in Ca_1‐x_Na_x_Ta_0.5_Ti_0.5_O_3_ upon Na substitution. However, even the CaTa_0.5_Ti_0.5_O_3_ sample (x=0.0) exhibits ferromagnetic behaviour, which is surprising given that the orthorhombic CaTiO_3_ and CaTaO_3_ phases, as well as cubic CaTiO_3_ of this composition, are typically non‐magnetic. This suggests that the ferromagnetic nature for x=0.0 might be due to the presence of the monoclinic Ca₂TiTa₂O₈ phase with a C2/c space group. Ca₂TiTa₂O₈ is known to possess ferromagnetic ordering with a magnetic moment of 2.0 μB/f.u. at room temperature.

A similar behaviour of magnetization is observed in other perovskite systems.[^26^, ^51^] A small quantity of the Ca₂Tia₂O₈ phase is presumed to be present in the x=0.0 sample. However, this phase is not distinctly evident in the XRD patterns, possibly due to the overlapping of Bragg's peaks with other phases.

It is observed that Hc for x=0.0 is 600 Oe which increased to 1300 Oe for x=0.4 samples, further increase in x decrease the Hc. The increase in coercivity may also be related to the particle size and grain boundaries which affect the domain wall movement. According to the Stoner–Wohlfarth theory coercivity is related to the anisotropy constant (*K)* through the following relation:^[52]^

(3) $$K=\;\frac{M_{s}\times H_{C}}{0.98}$$

where K is the anisotropy constant, *M*_s_ is saturation magnetization and *H*_c_ is coercivity. Similar to Hc; K is exhibiting highest value for x=0.4 (257 erg/cm^3^). The increase *K* indicates acceleration in the domain wall energy, and thus, it constrains free and easy domain wall motion. The variation in K is related to the Ms, Hc, particle size, domain wall motion, strain etc.[^53^, ^54^]

**Figure 5:**
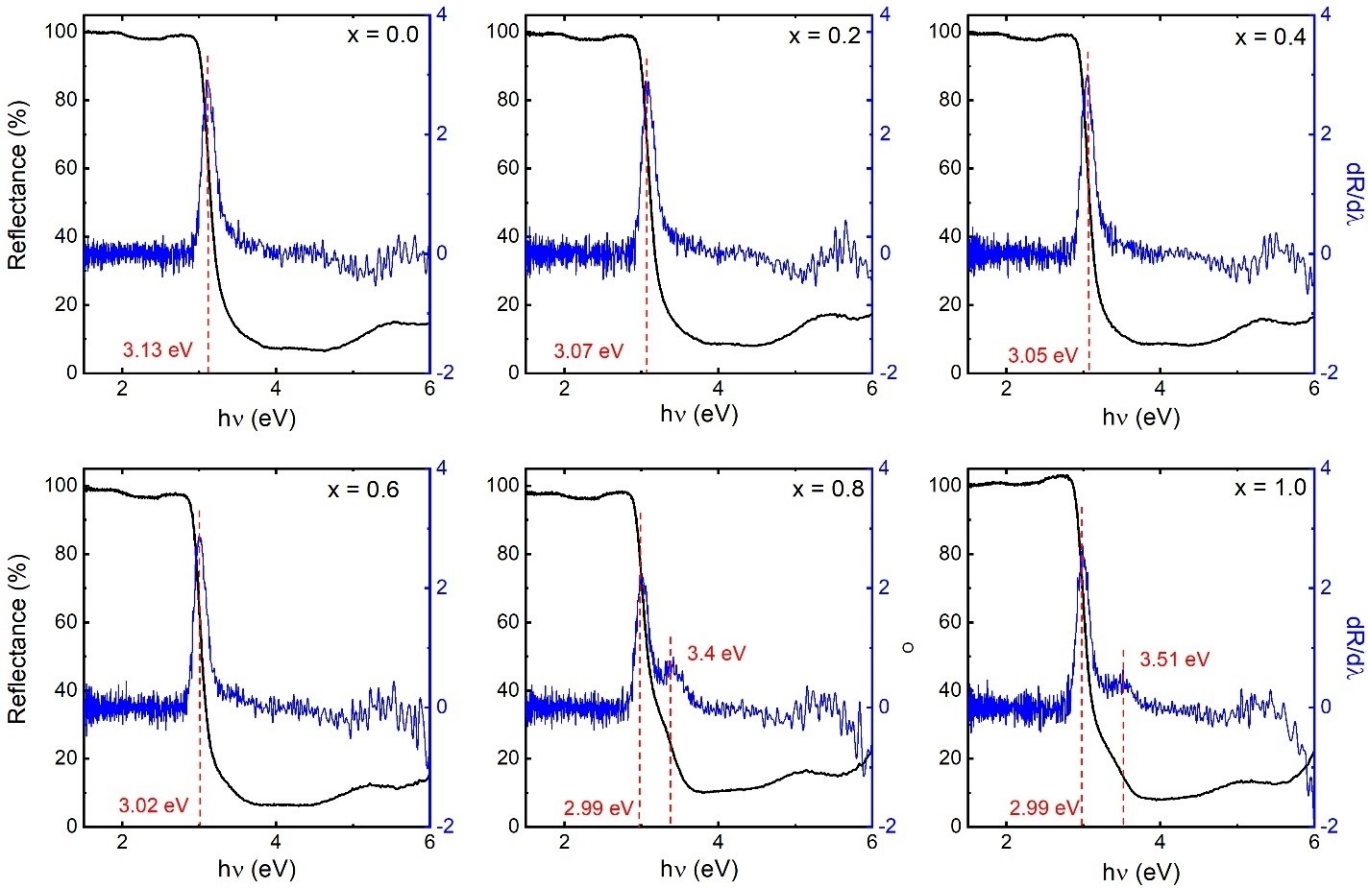
UV‐vis diffuse reflectance spectra and first derivative curves of the UV‐vis spectra.

Figure 5 depict the UV‐vis spectra of Ca_1‐x_Na_x_Ta_0.5_Ti_0.5_O_3_, along with the corresponding first derivative curves of the UV‐vis spectra. It is seen that the substitution of Na in to the CaTa_0.5_Ti_0.5_O_3_ obviously enhances the visible‐light absorption. The enhanced visible‐light absorption implies that the Ca_1‐x_Na_x_Ta_0.5_Ti_0.5_O_3_ photocatalyst can utilize photons more effectively.

**Figure 6:**
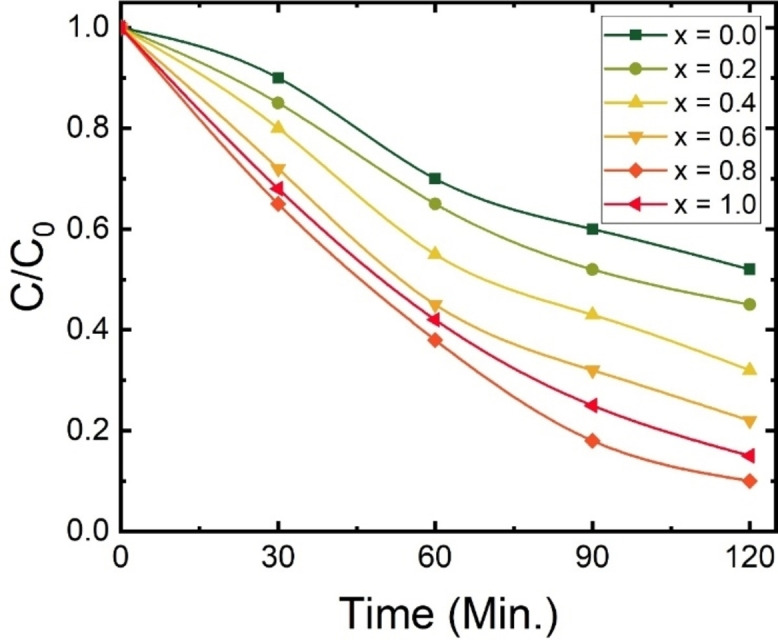
Time dependent photocatalytic properties of Ca_1‐x_Na_x_Ta_0.5_Ti_0.5_O_3_.

The photocatalytic activity of the synthesized Ca_1‐x_Na_x_Ta_0.5_Ti_0.5_O_3_ was investigated for its effectiveness in degrading Rhodamine B (RhB) dye under visible light exposure. The degradation rates observed in these experiments are presented in Figure 6. Initially, it was found that CaTa_0.5_Ti_0.5_O_3_ particles were able to degrade 49 % of the RhB solution within 120 minutes, indicating a moderate level of degradation capability. However, the efficiency of RhB degradation varied with the introduction of sodium (Na). Notably, the Ca_0.2_Na_0.8_Ta_0.5_Ti_0.5_O_3_ sample (x=0.8) exhibited a remarkable degradation rate of 90 % within the same time frame, marking the highest level of degradation efficiency observed in the study. The enhancement in photocatalytic activity with the addition of Na is attributed to improved utilization of visible light and the acceleration of electron separation and transmission processes. This improvement directly influences the photocatalytic degradation efficiency. The introduction of Na in various amounts demonstrated differing levels of photocatalytic activity. This variation is linked to the Na‐substituted CaTa_0.5_Ti_0.5_O_3_′s ability to absorb more visible light due to its narrower bandgap. However, an excessive substitution of Na (x=1.0) was observed to diminish the transmission of light energy, thereby impeding the photocatalytic reaction. Hence, an optimal level of Na substitution exists for maximizing photocatalytic efficiency. The UV‐Visible spectra, as shown in Figure 5, for samples with x=0.8 and 1.0, also revealed the presence of sub‐bands. These sub‐bands, along with surface modifications resulting from doping, contribute to a reduced rate of electron‐hole recombination. This reduction is a key factor enhancing the photocatalytic activity of these materials.[^38^, ^55^, ^56^]

## 3 Conclusions

In this comprehensive study, the structural, magnetic, and photocatalytic properties of Ca_1‐x_Na_x_Ta_0.5_Ti_0.5_O_3_ perovskites were systematically investigated. XRD, reveal a transition from tetragonal TiO_2_ structures to an orthorhombic CaTa_0.5_Ti_0.5_O_3_ structure, closely aligning with the orthorhombic structure of CaTiO_3_. This structural shift, precipitated by the Na‐for‐Ca substitution, leads to a decrease in Ca concentration, effectively curtailing the development of secondary phases and favoring the formation of a cubic phase within these perovskites. FE‐SEM and TEM analyses further provide insights into the nanocrystalline nature and particle size distribution, demonstrating significant structural changes with Na substitution. The magnetic characterization reveals an unexpected presence of ferromagnetic ordering within these perovskites, encompassing even those compositions historically regarded as non‐magnetic. This behavior suggests the presence of a monoclinic Ca₂TiTa₂O₈ phase with a C2/c space group, introducing ferromagnetic properties to the samples. Moreover, UV‐vis spectroscopy and photocatalytic activity tests reveal an enhanced visible‐light absorption and effective degradation of Rhodamine B dye under visible light, especially in samples with higher Na content. This enhancement is attributed to improved light utilization and accelerated electron separation, with an optimal Na substitution level identified for maximizing photocatalytic efficiency.

In conclusion, this study offers valuable insights into the influence of Na substitution on the structural, magnetic, and photocatalytic properties of Ca_1‐x_Na_x_Ta_0.5_Ti_0.5_O_3_ perovskites, highlighting its potential for various applications. The intricate interplay between structural changes, magnetic properties, and photocatalytic activity underlines the complexity and versatility of these materials.

## Conflict of Interests

The authors declare no conflict of interest.

4

## Data Availability

The data that support the findings of this study are available from the corresponding author upon reasonable request.
